# Supplementary material for: Quality of Life of People with Mobility-Related Disabilities in Sweden: A Comparative Cross-Sectional Study
Source: Int J Environ Res Public Health. 2022 Nov 16;19(22):15109. doi: 10.3390/ijerph192215109 (PMC9690284; doi:10.3390/ijerph192215109)
Supplement: Supplementary file 1 [file ijerph-19-15109-s001.zip › ijerph-1968610-supplementary-done.pdf]

International Journal of
Environmental Research and Public Health

MDPI

Table S1. ESS and SIS scores and wording.

	Question	Response Option	Transformation/Scoring	Source
Economic situation score, ESS	Do you have enough money to meet your needs?	1 (not at all); 2, 3, 4, 5 (completely)	No transformation, score 1-5	CBR indicator, lively hood
	Do you get to decide how to use your money?	1 (not at all); 2, 3, 4, 5 (completely)	No transformation, score 1-5	CBR indicator, lively hood
	Last year, has it happened that you must refrain from visiting a doctor or dentist, picking up medicines or aids because there is not enough money?	1 (Never),<br>2 (single occurrence),<br>3 (often),<br>4 (every month),<br>5 (several times a month)	Inverted, score 1-5	None
	Would you/your household be able to pay an unexpected expense of 12000 SEK within the month without lending money or asking for help?	Yes<br>No	Yes = 5<br>No = 1	Statistics Sweden
	During the last six months:<br>I have [SELECT OPTION] had enough money to be able to do the same things as my friends?	1 (Always),<br>2 (often),<br>3 (sometimes),<br>4 (rarely),<br>5 (never)	Inverted, score 1-5	WAYA study
Social inclusion score, SIS	Do you feel that other people respect you? For example, do you feel that others value you as a person and listen to what you have to say?	1 (not at all); 2, 3, 4, 5 (completely)	No transformation, score 1-5	CBR indicator, social
	Do you get to make decisions about the personal assistance you need (who assists you, what type of assistance, when to get assistance)?	1 (not at all); 2, 3, 4, 5 (completely)<br>6 (not applicable)	No transformation for 1-5<br>6 (NA) = 5<br>Score 1-5	CBR indicator, Social
	Do you get to make decisions about your personal relationships, such as friends and family?	1 (not at all); 2, 3, 4, 5 (completely)	No transformation, score 1-5	CBR indicator, Social
	Do you get to participate in artistic, cultural, or religious activities? (or could if you wanted to)	1 (not at all); 2, 3, 4, 5 (completely)	No transformation, score 1-5	CBR indicator, social
	Do you get to participate in recreational, leisure, and sports activities? (or could if you wanted to)	1 (not at all); 2, 3, 4, 5 (completely)	No transformation, score 1-5	CBR indicator, social
	To what extent do you know your legal rights?	1 (not at all); 2, 3, 4, 5 (completely)	No transformation, score 1-5	CBR indicator, social
	Do you get to make the big decisions in your life? For example, deciding who to live with, where to live, and how to spend your money?	1 (not at all); 2, 3, 4, 5 (completely)	No transformation, score 1-5	CBR indicator, empowerment
